# Monitoring the Skin Biophysical Parameters Among Coronavirus Patients for Three Days in a Row: a Preliminary Study

**DOI:** 10.2478/joeb-2022-0004

**Published:** 2022-05-20

**Authors:** Jalal M. Salih, Dindar S. Bari, Haval Y. Yacoob Aldosky

**Affiliations:** 1 University of Duhok, College of Science, Duhok, Kurdistan Region, Iraq; 2 University of Zakho, Faculty of Science, Zakho, Kurdistan Region, Iraq

**Keywords:** Coronavirus, oil, patients, pH, sebum, skin moisture

## Abstract

The coronavirus epidemic 2019 is spreading all over the world now. Several parameters are used to monitor the status of hospitalized patients; however, monitoring variations in biophysical properties of the skin has not been investigated yet. In this preliminary study, we seek to monitor skin biophysical parameters among coronavirus patients for three days in a row. Skin moisture, pH, sebum, and temperature during the three days were monitored in 30 coronavirus patients by using non-invasive portable instruments. Skin biophysical parameters were increased on the third day of monitoring compared to the first one. In addition, the increase in both skin moisture and temperature were statistically significant. According to the results of this preliminary study, skin biophysical parameters changed (increased) during the specified period in which the patients were monitored. However, changes in skin sebum content and pH were not significant. These skin parameters need to be further investigated until we know their indication ability for the health condition of coronavirus patients in clinical applications.

## Introduction

The skin is the largest multifunctional organ of the human body with a total surface area of about 2 m^2^. The skin is composed of a set of complex organs, which provide protection and sense functions. As a physical barrier, the skin protects the body from environmental threats such as temperature, pathogens, and mechanical, and chemical impacts by acting as a selective barrier. From a sensory standpoint, the skin has several receptors, which provide afferent information related to pain, touch, and temperature [1]. The skin has three layers; epidermis, dermis, and the subcutaneous layer (hypodermis). The stratum corneum is the uppermost part of the epidermis and consists of dead, keratinized cells.

Healthy skin can be characterized by its biophysical characteristics, such as skin moisture, sebum (oil) content, and pH. The normal level of skin (stratum corneum) moisture is influenced by various factors such as the amount of supplied water from the viable epidermis, dermis, and sebaceous glands, and also the stratum corneum's ability to accumulate water, and the volume of water lost through evaporation [2]. Moreover, the value of skin moisture can also be influenced by the type of consumed foods [3]. The skin sebum level is linked to individual characteristics and environmental conditions. Sebum production is dependent on the location, density, and activity of sebaceous glands [4]. Normal levels of sebum are associated with a high level of moisture [5]. Healthy skin has a pH value between 4.0 and 6.0. The normal pH value takes part in the maintenance of a normal level of skin moisture [2]. An increase in the skin pH leads to activation of cathepsins, breaking down of filaggrin, which decreases the natural moisturizing factor [6]. Also, an increase in transepidermal water loss is related to increased pH values [5].

Biophysical characteristics of the skin could also be a significant indicator of skin diseases. For instance, atopic dermatitis is associated with higher skin pH [7], and lower skin moisture and sebum [8]. In addition, a positive correlation between atopic dermatitis severity and pH values is observed in various studies [9]. Moreover, acne is related to increased skin pH [10], and sebum [11]. Therefore, understanding the biophysical properties characteristics of the skin might be useful in dealing with skin diseases with a proper approach.

To the best of our knowledge, no data are available on the skin biophysical parameters of patients with coronavirus. Therefore, in the present study, skin moisture, sebum and pH were measured to investigate the association between skin biophysical properties and coronavirus patients for three days in a row. Without any doubt, the body temperature, the primary test that is performed, of patients who had confirmed coronavirus symptoms was significantly higher than that of the unaffected controls. Therefore, this study hypothesized that an increase in skin temperature in coronavirus patients leads to altering its biophysical parameters such as skin moisture, sebum, and pH for three consecutive days of hospital stay.

## Materials and methods

### Study protocol and patients

A total of 30 coronavirus patients (14 males and 16 females), age range 27–90 years (mean 64 years) were enrolled in the study. All the recordings were performed at the coronavirus hospital in Duhok city under standard working conditions (T= 20-23 °C and relative humidity 40-60%).

Skin moisture, sebum content, pH, and temperature data were recorded from patients for three days in a row. It is worth noting that severe cases were not included in this study. All precautionary measures related to coronavirus were considered during the data collection.

### Instrumentation

Skin moisture, oil (sebum), pH, and temperature were recorded by using three different portable instruments. An SK-IV digital moisture monitor (Riuty, China) was employed to measure skin moisture and oil. It is based on bioimpedance technology and could measure the skin moisture within the range of 0-99.9%. Skin pH was measured by employing a pH meter with an accuracy of ±0.1 pH, using a high precision, corrosion resistant probe. Lastly, skin temperature was monitored by utilizing an infrared thermometer device (EFT-162-China).

### Statistical analysis

Differences among skin moisture, oil and pH recordings were statistically analyzed by using the one-way repeated analysis of variation (ANOVA) followed by post hoc multiple pairwise comparisons using Sidak correction. The statistical analyses were done by employing IBM SPSS Statistics and the 0.05 level of confidence was used to define statistical significance.

### Informed consent

Informed consent has been obtained from all individuals included in this study.

### Ethical approval

The protocol has been complied with all the relevant national regulations, institutional policies and in accordance with the Helsinki Declaration, and the study protocol was approved by the director of the coronavirus hospital in Duhok city.

## Results

### Skin moisture

**Fig.1:**
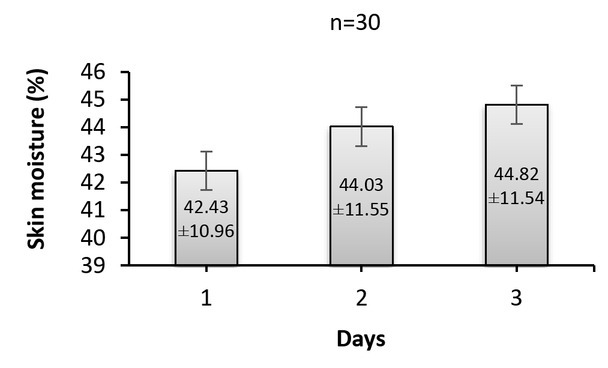
Skin moisture recorded from 30 patients during the three days.

Skin moisture data recorded during three days for all patients are presented in Figure 1. It can be seen that there are variations in the skin moisture of patients. In addition, on average, skin moisture was increased by comparing the second (44.03%) and third days (44.82%) to the first one (42.43%). Moreover, statistical analysis with ANOVA tests showed significant (*p*<0.05) differences between data of the three days, and also the data of the third day were significantly (*p*<0.05) different from the first and second days as indicated by post hoc multiple pairwise comparison using Sidak correction.

### Skin sebum content

**Fig.2:**
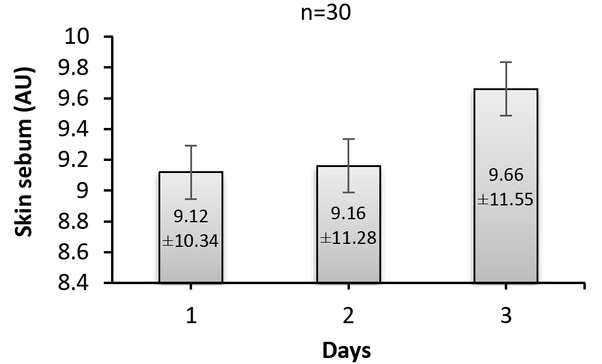
Skin sebum content recorded from 30 patients during the three days.

Shown in Figure 2 is the skin sebum content computed for each patient during the three days.

According to the data seen in the figure, skin sebum content values for the patients are slightly changed with respect to days. In addition, on average per 30 patients, skin sebum content is increased from 9.12 (on the first day) to 9.19 (on the second day), and 9.66 (on the third day). However, when the obtained data from over 30 patients were analyzed with ANOVA tests, non-significant (*p*>0.05) differences were observed over the three days.

### Skin pH

**Fig.3:**
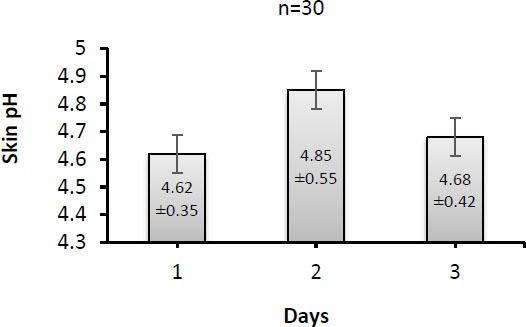
Skin pH recorded from 30 patients during the three days.

The results for skin pH with respect to the three days are presented in Figure 3. Variations in values of skin pH are also observed. Moreover, fluctuations are seen in average values of skin pH, but comparing the third day (4.68) to the first day (4.62), it is slightly increased. However, when the statistical analysis with post hoc pairwise multiple comparison tests was employed, non-significant (*p*>0.05) differences among the data for three days were obtained.

### Skin temperature

**Fig.4:**
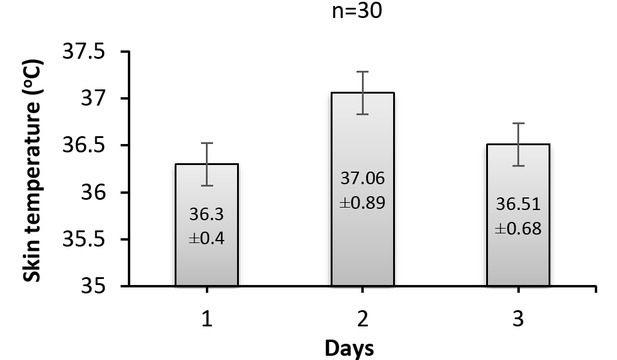
Skin temperature (^o^C) recorded from 30 patients during the three days.

Fluctuations are also seen (Figure 4) in the skin temperature of patients during the three days. In addition, highly significant (*p*<0.001) differences were found between skin temperature results recorded during the three days. Moreover, skin temperature levels monitored during the second day were significantly (*p*<0.001) different from those measured throughout the other days as indicated by post hoc pairwise multiple comparison tests.

## Discussion

This study aimed at recording biophysical parameters of the skin of coronavirus patients for three days in a row. According to the initial results, the biophysical characteristics of the coronavirus patients changed during the period of the study recordings.

Skin moisture, or the retention of water in the outermost layer of the skin known as the stratum corneum, which regulates skin inflammation, proliferation, and differentiation [12, 13], was significantly increased. This might be due to an increase in skin temperature and sweating, which consequently leads to increases in skin hydration. Jung *et al*. [14], reported that there is a positive correlation between skin temperature and skin moisture. Also, an increase in skin moisture could be related to elevated breathing rate or increased respiratory effort of the patients, as on average, the respiratory rate is elevated during diseases [15].

Small variations are obtained in the skin sebum content. According to the observed findings, skin sebum on average was slightly increased compared to the first day, but these results were statistically non-significant (*p*>0.05). One of the reasons is probably that increased body temperature led to an increase in sebum excretion rate. Cunliffe *et al*. [16], reported a significant correlation between skin temperature and the skin sebum excretion rate and showed that the skin sebum rate increased by 10% for each temperature rise of 1 °C. Also, Park *et al*. [17], linked the increased sebum secretion with the increase in body temperature. Jung *et al*. [14], also noted a positive relationship between skin temperature and sebum secretion. Also, there are other factors such as gender, ethnicity, hormones, and age [12] that are not considered in this study and could affect sebum secretion.

There were fluctuations in the skin pH of patients over the three days of monitoring. On the second and third days, it increased. The skin acidic milieu is necessary for epidermal permeability barrier homeostasis, restoration of the disrupted barrier, and the skin nonspecific antimicrobial defense [18, 19]. The reduction in skin pH on the third compared to the second day, may be due to increased body temperature and sweating. Jung *et al*. [14] showed that there is a negative correlation between skin temperature and skin pH. In addition, they reported that as the skin moisture increases, skin pH decreases [14].

Skin temperature monitoring plays an important role in the preliminary screening of coronavirus patients. Although on average skin temperature did not exceed the normal range, the temperature of patients on average rose significantly (*p*< 0.001) on the second day, then lowered in the third day. However, this does not mean that no patient had a high temperature, since several of the patients had an elevated skin temperature on the second day, which might be due to inflammation. In addition, it is reported that fever is one of the prominent clinical symptoms of novel coronavirus patients [20]. Moreover, the decrease in temperature (3^rd^ day) may be due to the patients being given antipyretic drugs as a part of the hospital treatment protocol. Generally speaking, when the skin temperature of a patient exceeds 37.3 °C, such as in this study it means that they have reached the fever standard.

The current study is the first that monitored the variations in biophysical properties of the skin for hospitalized coronavirus patients. Yet, two limitations must be considered. First, we tried to measure the variables for the first three days of symptoms appearing in patients infected with the coronavirus; however, controlling the admission of patients to the hospital as soon as symptoms appear is very difficult. Independent replication over longer periods with larger samples is needed to assess the strength of the directionality of the effects identified here. Second, we analyzed the data based on the provided treatment protocol at the hospital, and no information on the treatment provided to patients was obtained.

## Conclusions

Overall, we observed that skin moisture, sebum content, pH, and skin temperature increased over three days in a row of recordings. Changes in both skin moisture and temperature were significant, but changes in skin sebum and pH were non-significant. Skin biophysical parameters could be further investigated to be used as a useful indicator for the health conditions of coronavirus patients in clinical settings.
